# Outcomes and predictors of unplanned intensive care unit admission for pediatric trauma patients

**DOI:** 10.1016/j.sopen.2024.12.002

**Published:** 2024-12-18

**Authors:** Tyler Liang, Areg Grigorian, Robert Painter, James Jeng, Theresa Chin, Laura F. Goodman, Yigit S. Guner, Catherine Kuza, Jeffry Nahmias

**Affiliations:** a Division of Trauma, Burns, Critical Care & Acute Care Surgery, University of California Irvine, 101 The City Dr S, Orange, CA 92868, USA; b Children's Health of Orange County, 1201 W La Veta Ave, Orange, CA 92868, USA; c Department of Anesthesiology, Keck School of Medicine of the University of Southern California, Keck Hospital of USC, 1500 San Pablo Street, Los Angeles, CA 90033, USA

**Keywords:** Unplanned intensive care unit admissions, Pediatric trauma

## Abstract

**Background:**

Unplanned intensive care unit (ICU) admission (UIA) is associated with increased morbidity in adult trauma patients, however, is not well studied in pediatric trauma patients (PTPs). We sought to identify predictors of UIA, hypothesizing PTPs with UIA have increased odds of mortality.

**Methods:**

The 2017–2019 Trauma Quality Improvement Program (TQIP) database was queried for PTPs ≤16-years-old admitted to non-ICU level of care. Patients with UIA were compared to those without UIA. Multivariable logistic regression analysis was performed to determine predictors of UIA.

**Results:**

From 142,160 PTPs, 233 patients had UIA (<1 %). The UIA group had increased acute kidney injury (2.6 % vs 0 %, p < 0.001), length of stay (7 vs 2 days, p < 0.001), and mortality (1.3 % vs. 0.1 %, p < 0.001). Independent predictors of UIA included ureteral, esophageal, and brain injury (all p < 0.001).

**Conclusion:**

UIA for PTPs is rare but associated with increased complications and death. Significant predictors of UIA include ureteral, esophageal and brain injury.

## Highlights

• Unplanned intensive care unit admission (UIA) in pediatric trauma patients is associated with morbidity and mortality.
• Independent predictors of UIA in pediatric trauma patients include esophageal and ureteral injury.
• Understanding independent predictors of UIA in pediatric trauma patients may help stratify patients at risk for having a UIA.

## Introduction

Pediatric trauma is a leading cause of mortality among children, accounting for a significant portion of emergency department visits and hospital admissions [1]. Early identification and appropriate management of critically injured children are crucial for minimizing complications and improving outcomes. One key aspect of this process is determining the appropriate level of care for pediatric trauma patients (PTPs) upon admission, as incorrect triage of patients can lead to delayed intervention, increased complications, and higher mortality [[2], [3], [4], [5], [6], [7]].

In adult trauma populations, unplanned intensive care unit (ICU) admissions (UIA) are associated with increased morbidity and mortality [8,9]. In addition, adult UIA have been associated with increased length of stay (LOS) and risk of complications, including major abdominal surgeries [[2], [3], [4], [5],[8], [9], [10], [11], [12]]. Furthermore, up to 30 % of adult trauma ICU admissions are unplanned and some comorbidities such as alcoholism, cirrhosis, heart failure, hypertension, sepsis, and age have been identified as predictors of UIA [9,10,[12], [13], [14], [15]].

However, there is limited data available regarding UIAs in the pediatric trauma population, and the unique characteristics of PTPs require separate investigation. Identifying factors associated with UIA in PTPs could aid in better triage and allocation of resources. Current literature on pediatric UIAs has primarily focused on single-center studies, which may not capture the full range of variability in patient demographics, injury mechanisms, and healthcare settings. In addition, existing research has not identified specific injury patterns or clinical predictors that can be used to better triage PTPs.

Therefore, this study aims to use a national database to investigate the incidence, demographics, injury profiles, and outcomes of PTPs with UIA compared to those without UIA. Additionally, the study aimed to identify independent risk factors associated with UIA in this population, hypothesizing that specific injury patterns and clinical factors will be strongly associated with UIA in PTPs, which could be used to help triage PTPs and improve patient outcomes.

## Methods

This retrospective study was deemed exempt by our Institutional Review Board and a waiver of consent granted. A retrospective analysis of the Trauma Quality Improvement Program (TQIP) database was performed, selecting all PTPs 16 years of age or younger between 2017 and 2019 who were initially admitted to a non-ICU location. The primary outcome was UIA, defined by TQIP as patients having an unplanned admission to the ICU “after initial transfer to the floor and/or patients with an unplanned return to the ICU after initial ICU discharge” [16]. The reason for UIA is not available within the TQIP database. Patients with unplanned ICU re-admission (e.g., “bounce backs”) who initially were admitted to an ICU then transferred out of the ICU and then readmitted to an ICU, were excluded to ensure a more homogeneous study population, as this “bounce back” population likely has distinct complications and risk factors [9,11,[17], [18], [19], [20], [21], [22], [23], [24]]. Furthermore, our approach aimed to provide insights into the risk at the time of admission, which could facilitate different strategies for initial level of care triage based on this analysis. Secondary outcomes for this study included in-hospital complications and mortality.

Descriptive statistics were performed in the two comparison groups: patients with and without UIA. We collected demographics and vitals on arrival including hypotension (systolic blood pressure < 90 mm Hg), tachypnea (respirations >22 breaths per minute), tachycardia (heart rate > 120 beats per minute), mechanism of injury, and type of injury. Demographics included age, history of substance abuse, comorbidities, and sex. Comorbidities included diabetes and smoking. Mechanism of injury included fall, motor vehicle collision, and pedestrian struck. The injury profile included the injury severity score (ISS) as well as specific injuries including upper and lower extremity fractures, facial fractures, hemothorax, traumatic brain injury (TBI), spine fracture, spinal cord injury, lung injury, splenic injury, ureteral injury, esophageal injury, and small intestine injury. Complications included cardiac arrest, cerebrovascular accident (CVA), unplanned intubation, acute kidney injury (AKI), unplanned return to the operating room, length of stay (LOS), and death. A Mann-Whitney *U* test was used to compare continuous variables and chi-square was used to compare categorical variables between cohorts. Continuous data were reported as medians with interquartile range and categorical data were reported as percentages.

We then performed an analysis to identify independent associated risk factors for UIA. The degree of association between variables and UIA was performed using a multivariable logistic regression analysis, specifically employing a forward logistic regression model. The variables were chosen after review of the literature and consensus among authors [[2], [3], [4], [5],[8], [9], [10], [11], [12], [13], [14]]. We chose variables that were readily available shortly after patient arrival so that it would be a clinically useful model. The variables included mechanism of injury, vitals (hypotension, tachycardia >120 beats per minute, and respiratory rate >22 breaths per minute) and specific injuries including spinal cord injury, thoracic vessels, skull fracture, single rib fractures, pneumothorax, hemothorax, hemopneumothorax, lung injury, diaphragm injury, esophageal injury, pelvic fractures, spleen injury, liver injury, gallbladder injury, bile duct injury, pancreas injury, stomach injury, small intestine injury, colon injury, rectum injury, kidney injury, ureter injury, bladder injury, urethral injury, face fracture, upper extremity fracture, lower extremity fracture, traumatic brain injury, cervical fracture, general spine fracture. The identified independent predictors of UIA were reported as an odds ratio (OR) with 95 % confidence intervals (CI). All p values were two sided with a statistical significance level < 0.05. All statistical analyses were performed with IBM SPSS Statistics for Windows, Version 28 (Armonk, NY: IBM Corp).

## Results

### Demographics and injury profiles

From 142,160 PTPs initially admitted to non-ICU level of care, 233 patients had UIA (0.16 %). The UIA group was older (median age: 11 vs. 8 years old, p = 0.005) and had higher rates of diabetes (1.3 % vs. 0.3 %, p = 0.005), smoking (2.2 % vs 0.7 %, p = 0.006), and substance abuse (1.7 % vs 0.4 %, p = 0.001), compared to the no-UIA cohort. There was no difference with respect to sex or hypotension on arrival between cohorts. However, the UIA group exhibited higher rates of tachypnea (55.1 % vs. 46.5 %, p = 0.01) and tachycardia (32.9 % vs. 22.7 %, p < 0.001) upon arrival (Table 1).

**Table 1:** Demographics of pediatric trauma patients initially admitted to a non-intensive care unit (ICU) level of care, with and without an unplanned ICU admission.

Characteristic	Non-unplanned ICU admission (n = 141,927)	Unplanned ICU admission (n = 233)	p value
Age, year, median (IQR)	8 (9)	11 (11)	0.005
ISS, median (IQR)	4 (5)	9 (11)	<0.001
Male, n (%)	90,492 (63.8 %)	159 (68.2 %)	0.156
LOS, days, median, (IQR)	2 (1)	7 (7)	<0.001
Comorbidities, n (%)			
Diabetes	412 (0.3 %)	3 (1.3 %)	0.005
Smoking	949 (0.7 %)	5 (2.2 %)	0.006
Substance abuse	566 (0.4 %)	4 (1.7 %)	0.001
Vitals on admission, n (%)			
Hypotension (SBP < 90 mm Hg)	2745 (2.1 %)	8 (3.7 %)	0.106
Tachypnea (>22/min)	63,889 (46.5 %)	125 (55.1 %)	0.01
Tachycardia (>120/min)	31,451 (22.7 %)	75 (32.9 %)	<0.001

IQR = interquartile range, ISS = injury severity score, LOS = length of stay, SBP = systolic blood pressure, min = minutes.

In terms of injury profile, the UIA group less commonly sustained a fall (27.9 % vs 46.6 %, <0.001) mechanism of injury but more often suffered motor vehicle collisions (31.8 % vs. 18.6 %, p < 0.001) and pedestrian struck (8.2 % vs. 4.0 %, p < 0.001). All other mechanisms of injury were similar between cohorts (Table 2). Although the UIA group had a higher median ISS (9 vs. 4, p ≤0.001) they less commonly sustained upper extremity fractures (11.6 % vs. 29.3 %, p < 0.001) compared to the no-UIA group. However, the UIA group had increased rates of all other injuries, except facial fractures, hemothorax, and lower extremity fractures. Some of the most notable increased injuries in the UIA group included traumatic brain injury (19.3 % vs. 5.9 %, p < 0.001), spine fracture (12.4 % vs. 4.3 %), lung injury (17.2 % vs 4.7 %, p < 0.001), splenic injury (7.7 % vs. 1.8 %, p < 0.001), esophageal injury (0.4 %% vs 0.0 %, p < 0.001) and ureteral injury (0.9 % vs 0.0 %, p < 0.001) (Table 3).

**Table 2:** Mechanism of injury for pediatric trauma patients admitted to a non-intensive care unit (ICU) level of care, with and without unplanned ICU admission.

Mechanism	Non-unplanned ICU admission (n = 141,927)	Unplanned ICU admission (n = 233)	p value
Blunt injury			
Fall	66,094 (46.6 %)	65 (27.9 %)	<0.001
Pedestrian struck	5688 (4.0 %)	19 (8.2 %)	0.001
Bicycle	7466 (5.3 %)	9 (3.8 %)	0.339
Motorcycle	1979 (1.4 %)	4 (1.7 %)	0.675
Motor vehicle crash	26,332 (18.6 %)	74 (31.8 %)	<0.001
Penetrating injury			
Stab wound	2412 (1.7 %)	1 (0.4 %)	0.134
Gunshot wound	2863 (2.0 %)	5 (2.1 %)	0.889

**Table 3:** Injuries of pediatric trauma patients admitted to a non-intensive care unit (ICU) level of care, with and without unplanned ICU admission.

Injury	Non-unplanned ICU admission (n = 141,927)	Unplanned ICU admission (n = 233)	p value
Brain	8335 (5.9 %)	45 (19.3 %)	<0.001
Face	17,371 (12.2)	35 (15.0 %)	0.196
Cervical fracture	1097 (0.8 %)	8 (3.4 %)	<0.001
Spine fracture	6044 (4.3 %)	29 (12.4 %)	<0.001
Cervical spinal cord	291 (0.2 %)	6 (2.6 %)	<0.001
Rib fracture	2765 (1.9 %)	15 (6.4 %)	<0.001
Lung injury	6733 (4.7 %)	40 (17.2 %)	<0.001
Pneumothorax	3607 (2.5 %)	19 (8.2 %)	<0.001
Hemothorax	123 (0.1 %)	1 (0.4 %)	0.077
Hemopneumothorax	315 (0.2 %)	3 (1.3 %)	<0.001
Diaphragm	23 (0.0 %)	1 (0.4 %)	<0.001
Cardiac	86 (0.1 %)	0 (0.0 %)	0.707
Spleen	2551 (1.8 %)	18 (7.7 %)	<0.001
Ureter	16 (0.0 %)	2 (0.9 %)	<0.001
Kidney	1481 (1.0 %)	7 (3.0 %)	0.003
Esophagus	23 (0.0 %)	1 (0.4 %)	<0.001
Stomach	40 (0.0 %)	1 (0.4 %)	<0.001
Small intestine	446 (0.3 %)	8 (3.4 %)	<0.001
Colon	210 (0.1 %)	3 (1.3 %)	<0.001
Rectum	98 (0.1 %)	1 (0.4 %)	0.037
Kidney	1481 (1.0 %)	7 (3.0 %)	0.003
Upper extremity fracture	41,584 (29.3 %)	27 (11.6 %)	<0.001
Lower extremity fracture	30,211 (21.3 %)	57 (24.5 %)	0.237

### Outcomes and complications

Compared to the no-UIA group, the UIA cohort had increased unplanned intubation (7.9 % vs. 0.0 %, p < 0.001), AKI (2.6 % vs. 0.0 %, p < 0.001), cardiac arrest (1.7 % vs. 0.0 %, p < 0.001), and CVA (1.7 % vs. 0.0 %, p < 0.001). Additionally, the UIA group had a higher rate of unplanned return to the operating room (4.4 % vs. 0.1 %, p < 0.001) and death (1.3 % vs. 0.1 %, p < 0.001) (Table 4). The UIA group also had a longer median LOS (7 vs. 2 days, p < 0.001).

**Table 4:** Complications for pediatric trauma patients admitted to a non-intensive care unit (ICU) level of care, with and without unplanned ICU admission.

Complication	Non-unplanned ICU admission (n = 141,927)	Unplanned ICU admission (n = 233)	p value
Cardiac arrest	9 (0.0 %)	4 (1.7 %)	<0.001
Myocardial infarction	2 (0.0 %)	0 (0.0 %)	0.955
CVA	1 (0.0 %)	4 (1.7 %)	<0.001
Superficial SSI	16 (0.0 %)	0 (0.0 %)	0.872
Deep SSI	12 (0.0 %)	0 (0.0 %)	0.889
Osteomyelitis	5 (0.0 %)	0 (0.0 %)	0.928
Sepsis	5 (0.0 %)	4 (1.7 %)	<0.001
Pressure ulcer	34 (0.0 %)	1 (0.4 %)	<0.001
Unplanned intubation	6 (0.0 %)	18 (7.9 %)	<0.001
VAP	3 (0.0 %)	0 (0.0 %)	0.945
DVT	21 (0.0 %)	1 (0.4 %)	<0.001
Pulmonary embolism	6 (0.0 %)	1 (0.4 %)	<0.001
CAUTI	9 (0.0 %)	0 (0.0 %)	0.904
CLABSI	3 (0.0 %)	0 (0.0 %)	0.945
Acute kidney injury	11 (0.0 %)	6 (2.6 %)	<0.001
UROR	100 (0.1 %)	9 (4.4 %)	<0.001
Death	76 (0.1 %)	3 (1.3 %)	<0.001

CVA = cerebrovascular accident, SSI = surgical site infection, VAP = ventilator associated pneumonia, DVT = deep vein thrombosis, CS = compartment syndrome, CAUTI = catheter associated urinary tract infection, CLABSI = central line associated blood stream infection, UROR = unplanned return to operating room.

### Independent associated risk factors for unplanned ICU admission

On multivariable logistic regression analysis, independent associated risk factors for UIA included ureteral injury (OR 38.53, CI 6.67–222.43, p < 0.001), esophageal injury (OR 37.33, CI 4.92–283.17, p < 0.001), spinal cord injury (OR 14.39, CI 8.19–25.3, <0.001), small bowel injury (OR 5.47, CI 2.39–12.50, <0.001) and TBI (OR 3.90, CI 2.75–5.54, p < 0.001) (Table 5).

**Table 5:** Independent associated risk factors for unplanned intensive care unit admission in pediatric trauma patients.

Risk factor	OR	CI (95 %)	p-Value
Ureteral injury	38.525	6.673–222.432	<0.001
Esophageal injury	37.334	4.922–283.169	<0.001
Spinal cord injury	14.396	8.187–25.314	<0.001
Small intestine injury	5.474	2.397–12.502	<0.001
Traumatic brain injury	3.901	2.749–5.536	<0.001
Pancreatic injury	3.798	1.449–9.958	0.007
Splenic injury	2.861	1.686–4.855	<0.001
Cervical spine fracture	2.213	1.044–4.694	0.038
Multiple rib fractures	2.107	1.113–3.988	0.022
Lung injury	1.92	1.255–2.936	0.003
Pelvic fracture	1.872	1.089–3.219	0.023
Liver injury	1.81	1.024–3.200	0.041
Lower extremity fracture	1.649	1.194–2.279	0.002
Tachycardia	1.561	1.161–2.100	0.003

## Discussion

UIA represents a potential opportunity to evaluate under triage. This study represents a comprehensive analysis of PTPs with UIA utilizing a national database. The incidence of UIA was <1 %, but the associated complications and adverse outcomes are notably higher compared to PTPs without UIA. Furthermore, independent associated risk factors for UIA included ureteral injury and esophageal injury both with an over 37 times increased risk of UIA, as well as other risk factors such as spinal cord, small bowel and TBI.

Not surprisingly, pediatric UIA patients have higher rates of complications and morbidity, as this may be why they are transferred into an ICU. However, efforts to predict and prevent UIA in this population are underway and may prove helpful [6,7,15,25,26]. A retrospective single center study on pediatric patients requiring UIA by Krmpotic et al. showed that almost half of UIA patients had abnormal respiratory rates and a third had abnormal heart rates [26]. Although this study did not specifically study trauma patients, the rates were similar to the rates of tachypnea and tachycardia seen in pediatric UIA trauma patients in our study. Furthermore, they found that half of their non-trauma pediatric UIA patients had pre-existing medical conditions. While our PTPs did not have as many comorbidities, this similarly was more common in those who had an UIA and should be noted by admitting trauma surgeons when deciding level of admission care. In regard to PTPs specifically, Schrock et al. found that patients with UIA had higher ISS and were older [27]. They further identified ISS, initial disposition to the operating room and older age as independent risk factors for UIA. This was similar to our study which also showed that pediatric UIA patient were more likely to be older and have increased injuries. However, our study attempted to provide more granular injury data than ISS, which is often not available until patients are discharged. This included injuries that may be expected such as TBI, spinal cord injury and esophageal injury as well as others that are relatively common such as small bowel injury. In addition, this study found rarer injuries such as a ureteral injury to be associated with UIA. Hence, providers should acknowledge these injuries at higher risk for UIA and incorporate this into their decision making for initial level of care for PTPs. By incorporating these risk factors into the decision-making process, healthcare providers may improve the accuracy of triage and subsequently mitigate the adverse complications associated with UIA.

Admission of patients to the correct level of care is imperative as improper allocation is associated with increased rate of mortality and complications [[2], [3], [4],^12^]. Identifying patients at risk for UIA at the initial triage could help mitigate mis-triage of patients. The CRASH score created by Prado et al. sought to identify adult trauma patients at risk for UIA [13]. They found that alcohol use, cirrhosis, and mental disorder were associated with UIA. Predictors of UIA common between adult and PTPs in this study were multiple rib fractures, pelvic fractures, small intestine injury, spinal cord injury, lower extremity fracture, TBI, and cervical spine fracture. However, predictors of UIA in the pediatric population did not include chronic co-morbidities like those in the adult population. Also, as previously noted two of the strongest predictors for UIA in the pediatric population were ureteral and esophageal injuries, which were not so in the adult population. This highlights the unique differences between the two populations and reinforces the important point of pediatric trauma care, that children are not just little adults. Future research is needed to see if like the CRASH score, a pediatric triage tool may be developed to help prevent UIAs and thereby mitigate the increased rates of complications seen in a subset of high-risk children.

Unlike the retrospective single center studies by Rubano et al. and Mulvey et al., this study was derived from a large national database (TQIP) and thus provided a larger population needed to power the study [7,8]. However, there are inherent limitations with use of a national deidentified database such as missing data, miscoding, and misclassification [28]. In addition, there is heterogeneity of centers including children's hospitals and adult Level I/pediatric Level II centers included in the study, which have been shown to have some differences in management/outcomes [29,30]. Access to and staffing of the non-ICU and ICU units may vary across centers. Another significant limitation is that TQIP does not provide an indication for each UIA. This may have implications regarding the identification of risk factors in this study, which given the retrospective design cannot imply causality. In addition, timing of UIA from hospital arrival or evolution of injury was not provided in the TQIP database. Furthermore, this study had a low incidence of UIA within the pediatric trauma population, thus limiting the ability to create a scoring tool to identify UIA in pediatric patients. Finally, missing pertinent variables within TQIP include admission level of care such as an intermediate or step-down unit and vital signs following arrival to the trauma center.

## Conclusion

The incidence of PTPs who undergo an UIA is <1 %, however the rate of complications in this cohort is higher compared to patients who have no-UIA. Furthermore, this study identified esophageal, ureteral, and spinal cord injury as the strongest independent associated risk factors for UIA for PTPs. The identified associated risk factors for UIA, including specific injuries and clinical factors, should be considered during the triage process to improve the allocation of resources and potentially patient outcomes. Future research is needed to develop a risk prediction tool that can help better triage PTPs. Additionally, quality improvement efforts should integrate these risk factors into clinical decision-making processes, aiming to minimize the complications and adverse outcomes experienced by this vulnerable patient population.

## CRediT authorship contribution statement

**Tyler Liang:** Writing – review & editing, Writing – original draft, Formal analysis. **Areg Grigorian:** Writing – review & editing, Project administration, Data curation. **Robert Painter:** Writing – review & editing. **James Jeng:** Writing – review & editing. **Theresa Chin:** Writing – review & editing. **Laura F. Goodman:** Writing – review & editing. **Yigit S. Guner:** Writing – review & editing. **Catherine Kuza:** Writing – review & editing. **Jeffry Nahmias:** Writing – review & editing, Project administration.

## Ethical approval statement

IRB exempt.

## Funding

This research did not receive any specific grant from funding agencies in the public, commercial, or not-for-profit sectors.

## Declaration of competing interest

None.
